# Anxiety disorders and childhood exposure to emotional abuse: The mediating role of disgust

**DOI:** 10.1192/j.eurpsy.2021.229

**Published:** 2021-08-13

**Authors:** M. Innocenti, G. Santarelli, V. Gironi, V. Faggi, N. Giaquinta, L. Lucherini Angeletti, G. Castellini, V. Ricca

**Affiliations:** Human Health Sciences, University of Florence, Firenze, Italy

**Keywords:** disgust, childhood, Anxiety, emotional abuse

## Abstract

**Introduction:**

Several studies demonstrate that disgust, defined as a revulsion response aimed at distancing an individual from a potentially harmful or noxious stimulus, is linked to post-traumatic stress following sexual trauma even when accounting for associated fear and anxiety. One of the suggested mechanisms implicated in this association is a feeling of mental contamination. Recent neuroimaging studies demonstrated that exposure to contamination activates the insular cortex. In addition, disgust sensitivity correlates with the activation of the insular cortex.

**Objectives:**

We aimed to investigate the psychopathological role of the emotion of disgust in the developement of anxiety symptoms in patient with an history of abuse.

**Methods:**

We enrolled 84 patients admitted in Psychiatric Unit of Careggi with diagnosis of Anxiety Disorders. We administered to them: Zung Anxiety Scale (ZSAS), Childhood Trauma Questionnaire (CTQ), Disgust Propensity and Sensitivity Scale-revised (DPSS-r).

**Results:**

**Figure:**
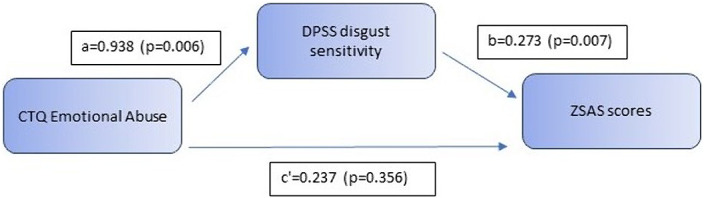

Results showed a significant mediation of the association between CTQ emotional abuse scores and total ZSAS scores via DPSS disgust sensitivity scores in patients with anxiety disorders (p=0.022). Total effect and indirect effect of emotional abuse on severity of anxiety symptoms were significant (total effect = 0.494; p=0.051, indirect effect: 0.256, p=0.022), while there was no significant direct effect from emotional abuse to anxiety symptoms in the total model (direct effect: 0.237, p=0.356). The model explained 18% of variance in anxiety symptomatology (R^2^=0.18).

**Conclusions:**

Such preliminary data suggest a possible mediating role of disgust in development and maintenance of childhood abuse-related anxiety, making it a potential target for psychotherapy.

**Disclosure:**

No significant relationships.

